# Reply to: Reported ultra-low lava viscosities from the 2021 La Palma eruption are potentially biased

**DOI:** 10.1038/s41467-023-42023-w

**Published:** 2023-10-16

**Authors:** J. M. Castro, Y. Feisel

**Affiliations:** Institute of Geosciences, University of Mainz, Mainz, Germany

**Keywords:** Geochemistry, Natural hazards

**replying to** G. Gisbert et al. *Nature Communications* 10.1038/s41467-023-42022-x (2023)

Castro and Feisel^1^ determined a range of viscosities of basanite magma erupted from Tajogaite volcano. Their measurements involve calibrated, hours-long experiments that reached steady-state viscosity plateaus. These hard data are useful for understanding eruptive phenomena that occurred within a specified timeframe (11/14/2021–11/25/2021) in November, 2021, directly on and near (<500 m) Tajogaite’s eruptive cone. As Castro and Feisel^1^ conclude, the freshly emergent magma’s odd hydrodynamic behavior was in part controlled by its ultralow viscosity. Castro and Feisel^1^ do not apply their results to processes at more distal locations, even though their experiments in the low temperature realm (e.g., T < 1130 °C) do provide additional constraints on the physical properties of extensively crystallized distal lavas.

In their comment, Gisbert et al.^2^ claim that the viscosity of freshly erupted magma at Tajogaite in November, 2021 was higher than the results of Castro and Feisel^1^ indicate. Their reasons include: (1) magma erupted at a lower T than geothermobarometers indicate; (2) magma contained less H_2_O than indicated by hygrometer-based estimates; (3) compositional differences between contemporaneous tephra and lava stemming from aerial fractionation; and 4) near-vent lavas were more crystalline than tephra. Here we address the accuracy and applicability of these comments to Castro and Feisel’s^1^ results.

## Temperatures

Clinopyroxene (cpx) microphenocryst – glass compositional data from tephra yield a range of permissible eruption temperatures (T ~ 1150–1200 °C)^1^. This range of average values incorporates cpx rims and cores, with values of rims defining the lower-T (1160–1170 °C) and cores the higher-T (~1200 °C) range. However, the range is also underpinned by cpx microphenocryst-rim and groundmass-microlite data that were provided in the Supplementary Information of Castro and Feisel^1^. These data, along with their measurement variance, indeed indicate high temperatures that overlap with values of microphenocryst cores. For example, cpx microlites yield a temperature spectrum (~1169 ± 26 °C) that overlaps with cpx-microphenocryst core values (~1200 ± 23 °C)^1^. Thus, Castro and Feisel’s^1^ results do indicate plausible eruption temperatures of ~1150–1200 °C.

Gisbert et al.^2^ contend that only the low, average cpx-rim temperature (1150 °C) is appropriate for viscosity calculations. They have ignored higher, cpx-rim and matrix-microlite-derived temperatures reported in Castro and Feisel^1^. However, these data are equally valid, and important for defining the *full* range of plausible eruption temperatures and estimated viscosities^1^.

Gisbert et al.^2^ assert that eruption temperatures at the vent may have been even lower than ~1150 °C. They cite a field-based lava temperature measurement (1140 °C). This datum is presented without indication of: 1) date and time; 2) location; 3) measurement method; and 4) instrument calibration and error. These details do not appear in the references they cite. Clearly, this information is necessary to validate the inference that lower eruptive temperatures are more realistic and applicable to the exact near-vent eruption phases addressed by Castro and Feisel^1^. Most importantly, the locations and timing of flows on which the cited temperature measurements were made must be comparable, if not identical, to the near-vent positions and times of the flows chronicled by Castro and Feisel^1^. This is because measurements made on lavas that are more distal than those described by Castro and Feisel^1^ will yield relatively lower temperatures due to the lavas’ longer run-out distances and travel times, and commensurate cooling. In the absence of field measurement details proving that near-vent lava was cooler, we consider this argument unjustified.

**Fig. 1:**
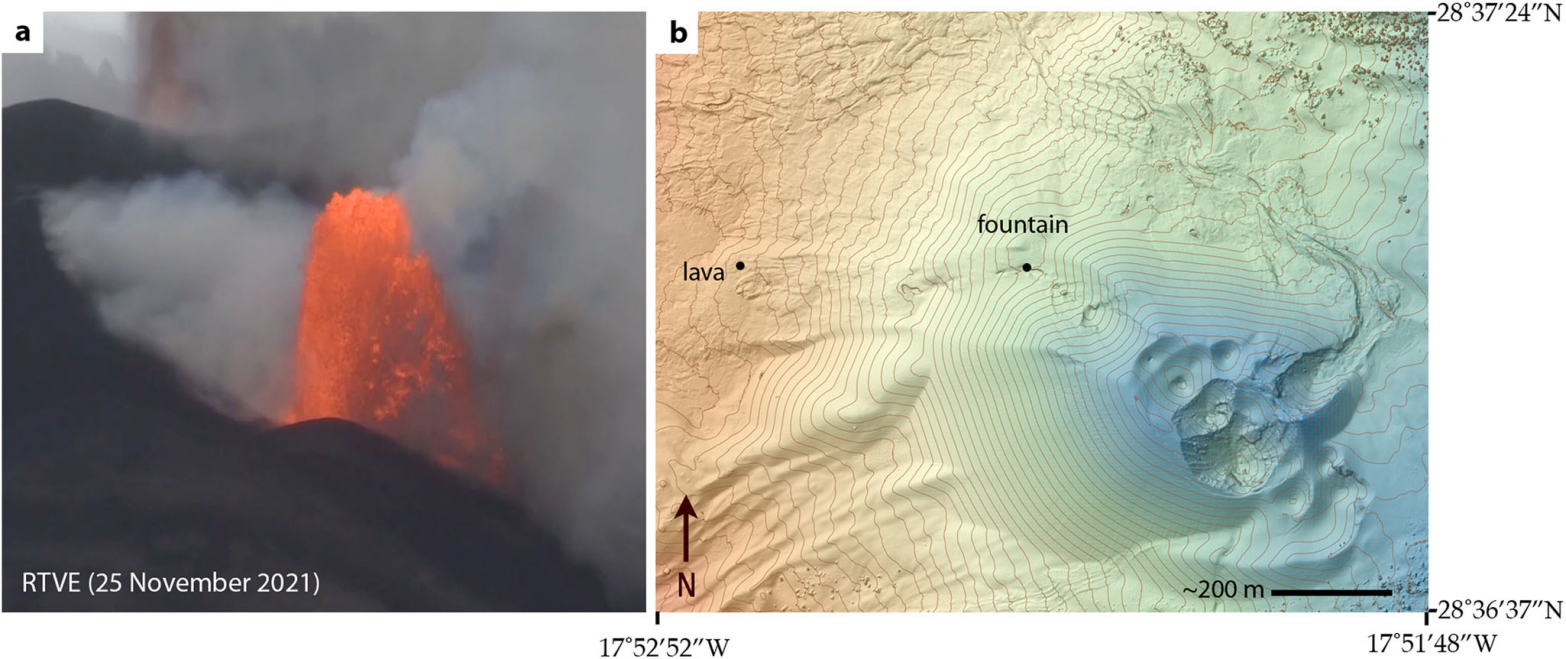
Fire fountaining at Tajogaite cone on 25 November, 2021. **a** Lava fountaining activity recorded at approximately 09:00 local time (presented by RTVE: Radiotelevisón Española)^14^. This lava fountain fed the rapid expansion of the main lava flow at Tajogaite^4^; these are same standing-wave-bearing lavas analyzed by Castro and Feisel^1^. Frame **b** presents a surface elevation model of the vent area^15,16^. Labels with black dots show the approximate positions of the lava fountain and flows studied by Castro and Feisel^1^. Note the close proximity of those lava flows to the source (~400 m). Maximum fountain heights (~80–120 m) were determined from various online videos of this activity, and comparisons of the fountain’s top with terrain-modeled elevations (frame **b**) of the active cone^15,16^. Contour lines in the digital surface model in frame **b** indicate relative scaling, and are distributed at an interval of 10 m. The maximum height of the fountain, along vesicularities (~20–40 vol.%) of erupted magma estimated from pyroclasts in Castro and Feisel^1^, implies overpressures at the fountain’s outlet of between ~1 and 2.5 MPa, following the formulations found in ref. ^9^. Such overpressures would result in significant retention of magmatic water (~0.5 wt.%)^10^ in the proximal lavas at the time of eruption, promoting their ultralow viscosity^1^.

Gisbert et al.^2^ suggest that the magma cooled during ascent in the conduit. Castro and Feisel’s^1^ data cannot thoroughly test this idea, however, it is reasonable to posit that the conduit system at the time of their study—approximately two months into the eruption—was thermally evolved. Magma ascent rates, if rapid, would additionally preclude significant conductive cooling upon ascent^3^. Strong fire fountaining (Fig. 1) that fed the rapidly moving near-vent lava flows on the morning of the 25 of November 2021^4^—the same lavas chronicled in Castro and Feisel^1^—is evidence of high magma ascent rates^5^. The small size of microlites and microphenocrysts (10’s to 100’s of µm)^1^, along with plausible growth rates (~0.03 µm s^−1^) constrained by experiments^1^, could also signal very short crystal growth and residence times in the conduit, indirectly implicating fast magma rise. Further to this point, if the relatively high implied storage pressures (7–10 kbar) and commensurate depths estimates (several to 10 s of km) that derive from the geothermometry on these crystals hold^1^, then very rapid ascent (several m’s per sec) is required to explain the crystals’ small size. Errors on pressure estimates are however large (±2 kbar)^1^, and thus, it is also possible that the microlites grew very late in the magma’s ascent path. In either case—deep versus shallow origin of microlites—the temperatures they record offer robust snapshots of the magma’s thermal properties during its supply and eruption, thereby defining the range of eruption temperatures (1150–1200 °C).

Finally, Gisbert et al.’s statement: “with the additional suggestion that eruptive temperatures of effusive lavas were higher”^2^, is inaccurate. Castro and Feisel^1^ made no statements or inferences to this effect. Rather, they provided detailed descriptions of eruptive activity that marked the formation of new vents emitting extremely fast and fluid lava.

## Magmatic water

Castro and Feisel^1^ estimated magmatic H_2_O contents (~0.8 wt. H_2_O) in order to define lower-bound viscosities in the framework of their experiments (see Fig. 6 in ref. ^1^). They concluded that in the case that the magma was even slightly hydrous^1^, viscosities of freshly emergent magma would be much lower (just tens of Pa sec) than dry magma.

Gisbert et al.^2^ indicate that H_2_O degassing should occur in equilibrium to low pressure. Therefore, lavas should emerge relatively dry and viscous. While this may be accurate for slow effusion driven by low magma decompression rates^6^, this was not the case during energetic phases of activity at Tajogaite, including the sudden increase in lava flux from the principle cone on 25 November 2021^4^. This episode of heightened activity produced a tall (~80–120 m; Fig. 1) fire fountain that fed the rapidly advancing, standing-wave-bearing, lava flows analyzed by Castro and Feisel (Fig. 2 in ref. ^1^). As lava fountains portend both elevated magmatic H_2_O and high flux^5,7^, this activity manifests a supply system in the throes of erupting magma that was likely ‘wetter’ than typical effusive basalts. As there are no direct measurements or sample-based analyses of H_2_O in products from the lava fountain, we must divine this magma’s water content another way. The maximum dissolved magmatic H_2_O depends on the lava fountain’s outlet pressure, which can be estimated from fountain height^8,9^. The 25 November 2021 fountain (Fig. 1) is consistent with outlet pressures ranging from ~1 to 2.5 MPa and, given the pressure dependence on H_2_O solubility^10^, dissolved H_2_O contents of ~0.42–0.53 wt.%. Thus, the eruption of higher H_2_O-content, ultralow-viscosity magma is warranted.

## The use of tephra instead of lava

Castro and Feisel^1^ present compositional data on tephra samples collected from proximal (<4 km) distal locations (~12.7 km). These tephras compositionally overlap with one another, and with glass inclusions^1^. Importantly, the tephras compositionally overlap with the most primitive (e.g., lowest SiO_2_) lava analyzed in ref. ^11^. Gisbert et al.^2^ contend that crystal winnowing could have offset tephra compositions from lava, thus making Castro and Feisel’s^1^ use of tephra sub-optimal for rheometry experiments. Winnowing should result in higher SiO_2_ in tephra compared to lava^11^. If aerial fractionation was important during the transport of tephra, then not only would Castro and Feisel’s tephra compositions^1^ plot in more-evolved space with respect to lavas^11^, but proximal and distal tephras should also be compositionally offset, owing to their disparate transport distances. Neither of these indicators exists in Castro and Feisel’s data^1^; we conclude that winnowing was not a factor, and Castro and Feisel’s use of tephras for rheometry experiments is both optimal and entirely justified. Finally, in the event that co-erupted lavas were indeed more primitive, then Castro and Feisel’s viscosities^1^ are *maximum* values.

## Crystal content and melt composition

Gisbert et al.^2^ speculate that the proximal lavas described by Castro and Feisel^1^ contained more crystals than tephras due to higher crystallization degrees and no crystal depletion from winnowing effects in the lavas. Their inference of higher near-vent lava crystallinity is based on textural measurements made on lava samples (e.g., LP-21-75; LP-21-82)^2^ that were erupted outside of the relevant eruption timeframe (14–25 November, 2021)^1^, and that were collected in very distal (>5 km) locations (e.g., LP-21-75; LP-21-77; LP-21-81)^2^, in contrast to the proximal positioning of flow and fountain activity addressed in Castro and Feisel (lavas ≤500 m from vent)^1^. Owing to these discrepancies, none of Gisbert et al.’s^2^ samples are one-to-one spatial and temporal proxies for the freshly emergent, supercritical lavas described by Castro and Feisel^1^. Their measurements on distal lava samples furthermore record advanced textural development resulting from very long (days) cooling and crystallization histories. Gisbert et al. provide no details or rationale for how they differentiate the effects of drawn-out surface flow crystallization from the inherent crystallinities that these lavas would have had at the time of their emergence from the vent^2^. Thus, their samples and measurements are a faulty basis for comparison to near-vent, rapidly quenched materials.

Most importantly, Gisbert et al.’s distal lava flow data^2^ cannot prove that the near-vent lavas were more crystalline than tephras erupted during the time period of activity covered by Castro and Feisel^1^. Video analysis of the activity during that time^1^ indicates vigorous, fast-moving lavas within a half kilometer of the vent (Fig. 1) and no evidence of people collecting samples or measuring these flows otherwise. Thus, the appropriate lava samples, ones that are sampled while still molten and then rapidly quenched, do not exist. We further note that lavas captured in the videos analyzed by Castro and Feisel had only been on the surface flowing for about a minute given their great velocities (~7–10 m sec^−1^)^1^ and proximity to the vent, which equates to a very abbreviated cooling and crystallization period. Castro and Feisel’s analysis indicates low tephra crystallinities (~6–16 vol.%)^1^ and no evidence of crystal loss in the form of a geochemical trend revealing an offset from primitive lavas^11^. We maintain that is is possible that lavas of equivalent low crystallinity to the tephras erupted at that time.

## Recalculating viscosities

Gisbert et al.’s viscosity estimates^2^ coincide with Castro and Feisel’s data in the low-temperature, high-crystallinity range (see Fig. 6 in ref. ^1^), validating some of Castro and Feisel’s ancillary findings^1^. However, Gisbert et al.’s assessment is only based on some, but not all of the data and eruption evidence in Castro and Feisel^1^, not to mention the new physical evidence we have provided in this reply (Fig. 1). By limiting their analysis to the parameter values they have selected (e.g., low eruption T, high crystallinity), Gisbert et al. have themselves introduced bias in their viscosity calculations, and thus restrict the relevance of their results. In particular, their analysis cannot explain the extreme dynamical aspects of Tajogaite’s near-vent activity that was characterized by the emergence of rapidly flowing and fountaining, extremely fluid basanite magma^1^. Effective hazard assessments of mafic eruptions^12,13^ must implement objective analysis of *all* potential parameter values and observed eruption phenomena. We conclude that Gisbert et al.’s analysis does not accurately portray the full spectrum of magmatic properties and eruption behaviors at Tajogaite^1^.

## Data Availability

All data analyzed in this work is included within this article or in ref. ^1^.
